# Vitamin C for patients with sepsis?

**DOI:** 10.3389/fmed.2024.1450091

**Published:** 2024-09-19

**Authors:** Harri Hemilä, Elizabeth Chalker

**Affiliations:** ^1^Department of Public Health, University of Helsinki, Helsinki, Finland; ^2^National Centre for Epidemiology and Population Health, Australian National University, Canberra, ACT, Australia

**Keywords:** critical illness, intensive care, mortality, rebound effect, scurvy, sepsis, statistics, time factors

The intensive care medicine rapid practice guideline (ICM-RPG) panel recently published a clinical practice guideline on the use of vitamin C for adult patients with sepsis. The panel recommended against its use (1). However, there are several factors which the panel seems not to have considered.

Specificity is important when looking at evidence for any intervention. If we want to determine whether vitamin C has an effect on sepsis, the comparison should be between vitamin C and a control, and not between vitamin C combined with other substances and a control (2). The panel considered that their most informative analysis was in their Supplementary Figure S1, but four of the six included trials administered vitamin C with other substances (1). This criticism is not speculative. A Korean cohort study by Jung et al. found that vitamin C alone was beneficial for patients with sepsis, whereas the combination of vitamin C and hydrocortisone was not (3). Trials of vitamin C and hydrocortisone combined are not appropriate surrogates for trials of vitamin C alone (2).

The panel “had more confidence in estimates of mortality at 90 days than at shorter time periods” though they were puzzled by “the potential difference between estimates of short- and long-term mortality”. However, the 90-day follow-up is misguided.

The largest trial in their Supplementary Figure S1 (1), the LOVIT trial, administered vitamin C for just 4 days (4). In the placebo group, there were 179 deaths by 90 days, which gives SD = 13 deaths, assuming a Poisson distribution. By the end of day 4, there were 51 deaths (5), which means that 72% of the deaths within 90 days occurred after vitamin C administration had ceased. Thus, 72% of the analyzed deaths cannot be attributed to the effects of ongoing vitamin C administration. The large SD at 90 days can hide substantial and genuine effects during and shortly after the 4-day ongoing vitamin C administration. This is also not speculative.

In the LOVIT trial, there was a significant increase in mortality in the vitamin C arm immediately after the vitamin was stopped, such that during days 5–7 there were 18 extra deaths in the vitamin C arm (5). This harm can be explained by the rebound effect, which has also been observed in a guinea pig study (5, 6). However, over the follow-up of the LOVIT trial, the short 3-day period during which the 18 extra deaths occurred is not apparent within the random variation over the 90 days. Some treatments such as vaccination cause long-lasting effects and long follow-ups are appropriate. However, there is no justification to assume similar long-term effects with vitamin C administration. The effect of vitamin C on mortality of patients with sepsis should be analyzed over the period of vitamin administration and shortly thereafter (Figure 1), and should not include several months without vitamin C.

**Figure 1:**
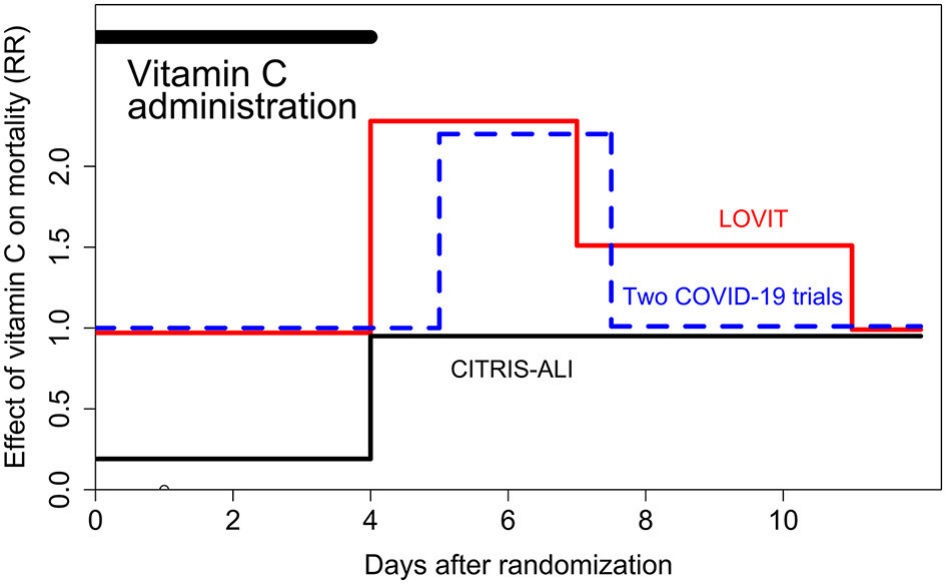
Change in mortality when 4-day intravenous vitamin C was abruptly terminated. RR = 1.00 indicates the level of the control group. This figure is based on the re-analyses of the LOVIT (4, 5), CITRIS-ALI (7, 8), and Two Harmonized Randomized Clinical Trials with COVID-19 patients (9, 10). In all trials, the vitamin C dose was 50 mg/kg body weight every 6 h (16 g/day for an 80 kg person) for 4 days. The CITRIS-ALI trial observed a significant reduction in mortality over the first 4 days, but thereafter the groups did not differ. The other two studies found no difference when vitamin C was administered, but after the abrupt termination of the vitamin there was a significant increase in mortality for a few days, after which the difference leveled off. In each study, there was significant difference in the RR before and after the end of the 4-day vitamin C administration (5, 8, 10) indicating that the abrupt termination of vitamin C had a harmful effect on mortality.

Moreover, the Korean study found that vitamin C was beneficial if the administration was ≥5 days, but ineffective if the administration was shorter (3). Although we need to be cautious about drawing treatment conclusions from cohort studies, the 4-day administration in the LOVIT trial may have been too short for patients with sepsis, one quarter of whom had ICU stays ≥ 12 days (4, 5).

The panel writes: “there may be patient populations where vitamin C has beneficial effects, such as … patients with confirmed low or low–normal plasma vitamin C levels” (1). However, no information was provided regarding the levels that are considered low or low-normal by the ICM-RPG panel. Usually, plasma vitamin C levels lower than 11 μM are considered deficient, but scurvy has also been observed with higher plasma levels (5, 11). In the LOVIT trial, 25% of patients had baseline vitamin C levels < 5.37 μM, which is half of 11 μM. One half of the LOVIT trial patients had plasma vitamin C levels below 12.38 μM (4). Should such patients be administered vitamin C routinely on the basis of low or low–normal plasma levels? The panel did not provide any guidance on this issue (1). If these patients need to be administered vitamin C on the basis of “confirmed low or low–normal plasma vitamin C levels” then it is unethical to randomize one quarter or one half of patients with low-vitamin C, similar to patients in the LOVIT trial, to the placebo group (5, 11).

There are numerous recent reports of patients suffering from scurvy and several of them were in the ICU (5, 10–20). Scurvy can cause dyspnea, edema, chest pain, and other pains, whereas gum pathology is not always present (5, 11). We are concerned that the ICM-RPG panel guideline may discourage the use of vitamin C for critically ill patients on the basis of statistically unsound analyses. Further research on vitamin C and sepsis is needed, but it is clear from the three trials which terminated 4-day vitamin C abruptly, that sudden termination is not appropriate if the patient is still critically ill (Figure 1).

## References

[1] Blaser AR Alhazzani W Belley-Cote E Møller MH Adhikari NKJ Burry L. Intravenous vitamin C therapy in adult patients with sepsis: a rapid practice guideline. Acta Anaesthesiol Scand. (2023) 67:1423–31. 10.1111/aas.14311 37500083

[2] Hemilä H Chalker E. Concerns with the revised Japanese recommendation for administering vitamin C to septic patients. J Intensive Care. (2023) 11:52. 10.1186/s40560-023-00702-2 37957677 PMC10641959

[3] Jung SY Lee MT Baek MS Kim WY. Vitamin C for ≥5 days is associated with decreased hospital mortality in sepsis subgroups: a nationwide cohort study. Crit Care. (2022) 26:3. 10.1186/s13054-021-03872-3 34983595 PMC8728994

[4] Lamontagne F Masse MH Menard J Sprague S Pinto R Heyland DK. Intravenous vitamin C in adults with sepsis in the intensive care unit. N Engl J Med. (2022) 386:2387–98. 10.1056/NEJMoa2200644 35704292

[5] Hemilä H Chalker E. Abrupt termination of vitamin C from ICU patients may increase mortality: secondary analysis of the LOVIT trial. Eur J Clin Nutr. (2023) 77:490–4. 10.1038/s41430-022-01254-8 36539454 PMC10115628

[6] Gordonoff T. Can water-soluble vitamins be over-dosed? Research on vitamin C (In German, translation available). Schweiz Med Wochenschr. (1960) 90:726–9. 10.5281/zenodo.11066294 13851245

[7] Fowler AA Truwit JD Hite RD Morris PE DeWilde C Priday A. Effect of vitamin C infusion on organ failure and biomarkers of inflammation and vascular injury in patients with sepsis and severe acute respiratory failure: the CITRIS-ALI randomized clinical trial. JAMA. (2019) 322:1261–70. 10.1001/jama.2019.11825 31573637 PMC6777268

[8] Hemilä H Chalker E. Reanalysis of the effect of vitamin C on mortality in the CITRIS-ALI trial: important findings dismissed in the trial report. Front Med. (2020) 7:590853. 10.3389/fmed.2020.590853 33117837 PMC7575729

[9] LOVIT-COVID Investigators Adhikari NKJ Hashmi M Vijayaraghavan BKT Haniffa R Beane A. Intravenous vitamin C for patients hospitalized with COVID-19: two harmonized randomized clinical trials. JAMA. (2023) 330:1745–59. 10.1001/jama.2023.21407 37877585 PMC10600726

[10] Hemilä H Chalker E. Rebound effect explains the divergence in survival after 5 days in a controlled trial on vitamin C for COVID-19 patients. Front Med. (2024) 11:1391346. 10.3389/fmed.2024.1391346 38841576 PMC11151746

[11] Hemilä H de Man AME. Vitamin C deficiency can lead to pulmonary hypertension: a systematic review of case reports. BMC Pulm Med. (2024) 24:140. 10.1186/s12890-024-02941-x 38504249 PMC10949735

[12] Baluch A Landsberg D. Scurvy in the intensive care unit. J Investig Med High Impact Case Rep. (2021) 9:23247096211067970. 10.1177/23247096211067970 34939441 PMC8721699

[13] Penn EH Olenchock BA Marston NA. A shocking deficiency. Circulation. (2019) 140:613–7. 10.1161/CIRCULATIONAHA.119.040894 31403849 PMC6812570

[14] Alnaimat S Oseni A Yang Y Melvani V Aronson A Harris K. Missing vitamin C: a case of scorbutic cardiac tamponade. JACC Case Rep. (2019) 1:192–6. 10.1016/j.jaccas.2019.07.006 34316783 PMC8301525

[15] Bennett SE Schmitt WP Stanford FC Baron JM. Case 22-2018: a 64-year-old man with progressive leg weakness, recurrent falls, and anemia. N Engl J Med. (2018) 379:282–9. 10.1056/NEJMcpc1802826 30021092 PMC6060279

[16] Doll S Ricou B. Severe vitamin C deficiency in a critically ill adult: a case report. Eur J Clin Nutr. (2013) 67:881–2. 10.1038/ejcn.2013.42 23549202

[17] Holley AD Osland E Barnes J Krishnan A Fraser JF. Scurvy: historically a plague of the sailor that remains a consideration in the modern intensive care unit. Intern Med J. (2011) 41:283–5. 10.1111/j.1445-5994.2010.02413.x 21426466

[18] Kieffer P Thannberger P Wilhelm JM Kieffer C Schneider F. Multiple organ dysfunction dramatically improving with the infusion of vitamin C: more support for the persistence of scurvy in our “welfare” society. Intens Care Med. (2001) 27:448. 10.1007/s001340000830 11396297

[19] Meisel JL McDowell RK. Case 39-1995: a 72-year-old man with exertional dyspnea, fatigue, and extensive ecchymoses and purpuric lesions. N Engl J Med. (1995) 333:1695–702. 10.1056/NEJM199512213332508 7477224

[20] Raynaud-SimonACohen-BittanJGouronnecAPautasESenetPVernyM. Scurvy in hospitalized elderly patients. J Nutr Health Aging. (2010) 14:407–10. 10.1007/s12603-010-0032-y20617280

